# Dr Jekyll and Mr Hyde: a strange case of 5-ethynyl-2′-deoxyuridine and 5-ethynyl-2′-deoxycytidine

**DOI:** 10.1098/rsob.150172

**Published:** 2016-01-06

**Authors:** Anna Ligasová, Radek Liboska, David Friedecký, Kateřina Mičová, Tomáš Adam, Tomáš Oždian, Ivan Rosenberg, Karel Koberna

**Affiliations:** 1 Institute of Molecular and Translational Medicine, Palacký University in Olomouc, Olomouc 77900, Czech Republic; 2 Institute of Organic Chemistry and Biochemistry, The Czech Academy of Sciences, v.v.i., Prague 16610, Czech Republic

**Keywords:** cytidine deaminase, dCMP deaminase, 5-ethynyl-2′-deoxyuridine, 5-ethynyl-2′-deoxycytidine, DNA replication

## Abstract

5-Ethynyl-2′-deoxyuridine (EdU) and 5-ethynyl-2′-deoxycytidine (EdC) are mainly used as markers of cellular replicational activity. Although EdU is employed as a replicational marker more frequently than EdC, its cytotoxicity is commonly much higher than the toxicity of EdC. To reveal the reason of the lower cytotoxicity of EdC, we performed a DNA analysis of five EdC-treated human cell lines. Surprisingly, not a single one of the tested cell lines contained a detectable amount of EdC in their DNA. Instead, the DNA of all the cell lines contained EdU. The content of incorporated EdU differed in particular cells and EdC-related cytotoxicity was directly proportional to the content of EdU. The results of experiments with the targeted inhibition of the cytidine deaminase (CDD) and dCMP deaminase activities indicated that the dominant role in the conversion pathway of EdC to EdUTP is played by CDD in HeLa cells. Our results also showed that the deamination itself was not able to effectively prevent the conversion of EdC to EdCTP, the conversion of EdC to EdCTP occurs with much lesser effectivity than the conversion of EdU to EdUTP and the EdCTP is not effectively recognized by the replication complex as a substrate for the synthesis of nuclear DNA.

## 1. Introduction

5-Ethynyl-2′-deoxyuridine (EdU) and 5-ethynyl-2′-deoxycytidine (EdC), representing analogues of 2′-deoxyuridine and 2′-deoxycytidine, respectively, were tested as substances with an anti-viral effect during the 1980s [1,2]. However, presently, their use is primarily connected with the detection of cellular replicational activity [3–7]. EdU was used for the detection of replicational activity for the first time in 2008 [7], EdC in 2011 [5].

The visualization of both modified nucleosides is mostly performed by click chemistry, the copper (I)-catalysed reaction between the ethynyl group of the nucleoside and azido group of the fluorochrome [5,7]. Until the use of EdU and EdC, the dominating nucleoside used for the analysis of DNA synthesis was 5-bromo-2′-deoxyuridine (BrdU). Its detection is based on the specific antibodies and steps enabling reaction of BrdU in DNA with antibodies [8–13]. The advantage of EdU/EdC compared to BrdU is the fact that the visualization of EdU/EdC does not require the specific steps necessary for the detection of BrdU. These steps often interfere with the detection of other cellular components (e.g. [14]). The introduction of EdU and EdC facilitated co-localization studies, e.g. with proteins, and accelerated the whole procedure (e.g. [15]).

On the other hand, the use of EdU and EdC is complicated by their cytotoxicity [16–22]. Although it was shown that the EdC toxicity is lower than that of EdU [5], the reason was not known. In the case of EdU, its toxicity is directly related to the extent of the incorporation of EdU into DNA. The presence of EdU in a DNA strand may result in the induction of interstrand cross-links [19]. The EdU toxicity is also enhanced by the fact that its 5′-monophosphate (EdUMP) inhibits thymidylate synthase [19,23]. As thymidine (dT) is a direct competitor of EdU during DNA synthesis, inhibition of thymidylate synthase in the presence of EdU also increases EdU incorporation into DNA and consequently its toxicity [19]. Moreover, this inhibition can lead to an imbalance in the nucleotide pool and subsequently to the impairment of DNA replication [19].

In the case of EdC, such effects were not described. However, the deamination of EdC by cytidine deaminase (CDD) and possible deamination of EdC monophosphate (EdCMP) by dCMP deaminase (DCTD) produce EdU and EdUMP, respectively. Already in 1985, Balzarini *et al.* [24] showed the stimulation effect of EdC on the growth of a thymidylate synthase-deficient murine mammary carcinoma cell line. As the stimulation effect was suppressed by the CDD inhibitor tetrahydrouridine and also by the CDD and DCTD inhibitor 2′-deoxytetrahydrouridine, the authors supposed that EdC is transformed to EdU which is incorporated into DNA. However, it was unclear how general this phenomenon is and what the effectivity of such a conversion is. In this respect, Qu *et al.* [5] interpreted the results of experiments focused on the EdU and EdC toxicity in several cell lines as proof that EdC follows the EdC → EdCMP → EdCDP → EdCTP pathway as a major metabolic pathway. It is supposed that the cellular deaminases are involved in the inactivation of drugs based on the 2′-deoxycytidine analogues (e.g. cytarabine and gemcitabine [25]). From this point of view the pair EdU and EdC are an interesting model system with the possibility of quick visualization of the incorporated nucleosides. In this respect, we have shown here that one of the anti-bromodeoxyuridine monoclonal antibodies that exhibits high affinity to EdU [26] does not effectively react with EdC.

In the study presented here, we focused on the efficiency of the conversion of EdC to EdU and particular steps leading to this conversion. Concurrently, we followed the toxicity of both nucleosides and tested the possibility that the toxicity is directly connected with the conversion of EdC to EdU.

Overall, the results obtained clearly showed that EdC and its metabolites are a substrate of a whole range of enzymes in the pathway leading to the production of EdCTP as well as in the opposite pathway leading to the degradation of EdCTP. Our results also indicate that the deamination activity mediated by CDD plays only a marginal role in the effective protection of cells from the EdC incorporation in HeLa cells. On the other hand, this activity substantially contributes to EdC toxicity due to the gradual conversion of EdC to EdUTP followed by the incorporation of EdU into DNA. In this respect, CDD paradoxically allows the use of EdC as a replicational marker, and concurrently, fundamentally contributes to its toxicity.

## 2. Material and methods

### 2.1. Cell cultures

Human HeLa cells (cervix, adenocarcinoma), 143B PML BK TK cells (bone, osteosarcoma, contains a herpes simplex virus type 1 thymidine kinase (hsv-1 TK+) plasmid; 143B), A549 cells (lung, carcinoma), U2OS cells (bone, osteosarcoma) and HCT116 cells (colon, colorectal carcinoma) were used. The cell lines were cultivated in an appropriate culture media (for more details, see the electronic supplementary material, S1). The cells were cultured on coverslips (12 mm in diameter) in a Petri dish or in 96-well plates (Orange Scientific) at 37°C in a humidified atmosphere containing 5% CO_2_.

### 2.2. MTT assay

The MTT assay was performed according to reported studies [12,19,27]. Briefly, the cells were seeded at the density of 5 × 10^3^ cells per well in 96-well plates and incubated for 24 h. The tested nucleosides were added to the culture media for 48 h. Serial fivefold dilutions of EdU and EdC were used starting at a 0.0032 µM concentration and ending at a 250 µM concentration. Then, the culture media were exchanged for nucleoside-free media for an additional 72 h. The freshly prepared 1 mM 3-(4,5-dimethylthiazol-2-yl)-2,5-diphenyltetrazolium bromide (MTT, ThermoFisher Scientific) was added for 3 h. The culture media were removed and DMSO was added to each well for 10 min at 37°C and 300 r.p.m. in a Thermomixer chamber (Eppendorf). Absorbance was measured using a PerkinElmer EnVision Plate Reader (Perkin Elmer) at 540 nm.

### 2.3. Inhibition of CDD and DCTD activity

CDD- and DCTD-specific and control siRNAs were purchased from Santa Cruz Biotechnology. We followed the protocol recommended by the supplier (for more details, see the electronic supplementary material, S2). The HeLa cells were treated with 50 nM siRNA. We used siRNAs against CDD and DCTD, consisting of pools of three target-specific 19–25 nt siRNAs. After transfection, the cells were incubated with either 10 µM EdU or 10 µM EdC for 2 h, fixed, permeabilized and the incorporated EdU/EdC were visualized by a click reaction or the cells were lysed and prepared for a western blot analysis.

In the case of the inhibition of CDD activity, we also used the CDD inhibitor tetrahydrouridine (THU) [24]. We incubated HeLa cells first with 10 µM THU for 30 min followed by the incubation with 10 µM EdC or EdU together with 10 µM THU for 2 h. The incorporated EdU was visualized by a click reaction.

### 2.4. *In situ* detection of EdU/EdC

After incubation of the cells with EdU or EdC, they were fixed with 2% formaldehyde and permeabilized with 0.2% Triton X-100 if not stated otherwise.

The click reaction was used for the detection of EdU/EdC in nuclear DNA [7,26]. We used the kit containing Alexa Fluor 488 azide and followed the manufacturer's protocol (ThermoFisher Scientific).

For exclusive EdU detection in nuclear DNA, we used an anti-bromodeoxyuridine antibody (clone B44, Becton Dickinson, primary antibody). In this case, EdU was revealed in the DNA structure using copper(I) ions [12,13] followed by a reaction with the anti-bromodeoxyuridine antibody supplemented with exonuclease III (1 U µl^−1^, ThermoFisher Scientific) and an antibody conjugated with Alexa Fluor 488 fluorochrome (Jackson ImmunoResearch, secondary antibody).

The nuclear DNA was stained by DAPI (10 µM, 30 min; room temperature).

### 2.5. Run-on replication

The HeLa cells cultured on the coverslips were quickly rinsed on three drops (approx. 100 µl each) of PBS followed by one drop of a mixture of D-buffer and PBS (1 : 1) and then three drops of D-buffer. Subsequently, the cells were incubated on a drop of 0.05% Triton X-100 in D-buffer for 3 min followed by a quick rinse on three drops of D-buffer. All the steps were performed on ice. The D-buffer contained 50 mM Tris–HCl, pH 7.2, 10 mM MgCl_2_, 100 mM KCl, 160 mM sucrose, 4% polyvinylpyrrolidone (average molecular weight 10 000), 1 mM dl-dithiothreitol (DTT) and 10% glycerol. DNA labelling was performed in the D-buffer containing 10 mM dATP, dCTP, dGTP and EdUTP or in the solution of D-buffer and 10 mM dATP, dTTP, dGTP and EdCTP for 20 min at 37°C. The next steps were performed at room temperature. The cells were washed on the drops of D-buffer and PBS (1 : 1) and drops of D-buffer, fixed with 2% formaldehyde for 10 min and permeabilized with 0.2% Triton X-100 for 10 min. After a wash with PBS, the incorporated nucleotides were detected using a click reaction.

### 2.6. Hypotonic treatment

The hypotonic treatment of HeLa cells was performed according to Koberna *et al.* [28]. Briefly, the cells were quickly rinsed with the 1× KHB buffer (30 mM KCl, 10 mM HEPES, pH 7.4) and incubated either with 0.4 mM EdUTP or EdCMP or EdCDP or EdCTP in 1× KHB for 10 min. In some experiments, 0.2 mM thymidine 5′-triphosphate (dTTP) was added to the hypotonic solution. Then, culture medium was added and the cells were incubated at 37°C in a humidified atmosphere containing 5% CO_2_. After 30 min, the cells were fixed. EdU/EdC were detected using either a click reaction or with the primary mouse anti-bromodeoxyuridine antibody clone B44.

### 2.7. Determination of EdU and EdC concentrations in cellular DNA

We incubated cells with either 10 µM EdU or 10 µM EdC for 24 h. The control cells were incubated without any modified nucleosides. After incubation, the DNA was isolated from approximately 1 × 10^6^ cells using a commercial isolation kit (MagJET Genomic DNA kit, ThermoFisher Scientific). The precipitated and denatured DNA (see electronic supplementary material, S3) was cleaved using P1 nuclease (3 × 90 min, 37°C), phosphodiesterase I from *Crotalus adamanteus* venom (2 × 60 min, 37°C) and alkaline phosphatase from *Escherichia coli* (2 × 60 min, 37°C). The nucleoside concentrations were evaluated from the corresponding peaks area (recorded at 260 nm UV) obtained from HPLC analysis and from pertinent extinction coefficients. The standard values of the extinction coefficients were used for nucleosides. The concentration of nucleoside analogue EdU was calculated from its peak area measured at 289 nm UV; the extinction coefficient of 12 000 l mol^−1^ cm^−1^ was used in this case. The HPLC analyses were performed on reversed phase columns (LUNA Phenomenex, C18) on a Waters Alliance chromatograph. The linear gradient of acetonitrile concentration in a triethylammonium carbonate (TEAB) buffer was chosen so as to ensure good separation of the nucleosides, particularly EdC, EdU and dT (gradient from 0.05 M TEAB to 10% acetonitrile in 0.05M TEAB over 20 min). The retention times for nucleosides and nucleoside analogues (EdC and EdU) were calibrated with the standards.

### 2.8. Western blot

HeLa cells were treated either with CDD siRNA or DCTD siRNA, or control siRNA for 48 h. The cells were lysed with RIPA lysis buffer (150 mM sodium chloride, 1% NP-40, 0.5% sodium deoxycholate, 0.1% SDS, 50 mM Tris, pH 8.0) on ice for 30 min and the lysates were centrifuged at 20 000*g* and 4°C for 10 min. The protein content was measured using the BCA assay (Sigma Aldrich). For SDS-PAGE electrophoresis, 5 µg of the total protein was resolved by SDS-PAGE at a constant voltage of 100 V. The proteins were then transferred to a nitrocellulose membrane (0.2 µm pore size, Bio-Rad) using the TransBlot Turbo semi-dry system (Bio-Rad). The membrane was blocked for 1 h in 5% non-fat dry milk in TBS/T (Tris-buffered saline with 0.1% Tween-20) and incubated with primary antibodies against CDD and DCTD (both from Santa Cruz Biotechnology) and β-actin (Sigma Aldrich) in 5% BSA and TBS/T overnight at 4°C with agitation. Then, the membranes were washed with TBS/T and incubated with peroxidase-labelled secondary antibodies. The membranes were washed and incubated briefly with Luminata Forte peroxidase substrate (Merck). The chemiluminiscence was collected by an HCD camera (Li-Cor Odyssey). The band intensities were then normalized to their respective actin band. The data were evaluated using Microsoft Excel software. The measurements were performed in five repetitions.

### 2.9. Biotin-labelled EdC and EdU preparation and its use for the analysis of anti-bromodeoxyuridine antibody reactivity

Biotin-labelled EdC and EdU were synthesized and the analysis of antibody reactivity with EdU and EdC was performed according to the described procedure ([26]; for more details see the electronic supplementary material, S4). The Reacti-Bind™ streptavidin-coated high binding capacity black 96-well plates (ThermoFisher Scientific—Pierce) were washed with a Tris buffer (25 mM Tris–HCl, pH 7.2; 150 mM NaCl; 0.1% BSA; 0.05% Tween-20) and incubated with 1 nmol of the prepared ligand per well (100 µl of 10 µM solution; 2 h at room temperature). After incubation, the well plates were washed with Tris buffer and incubated with the primary anti-bromodeoxyuridine antibody (clone B44, 1 : 10, in Tris buffer, 30 min, 24°C, 950 r.p.m.), washed again with Tris buffer and incubated with an Alexa Fluor 488 anti-mouse antibody (1 : 100 in Tris buffer, 30 min, 24°C, 950 r.p.m.). The signal was measured using a PerkinElmer EnVision Plate Reader (Perkin Elmer). The final graphs were made in Microsoft Excel. The measurements were performed for three independent experiments.

### 2.10. Nucleotide pools analysis

HeLa or 143B cells were incubated either with 10 µM EdU or 10 µM EdC for 4 h or without any treatment (control). In the case of hypotonic treatment, the HeLa cells were treated according to Koberna *et al.* [28]: the cells were quickly rinsed with 1 × KHB buffer and incubated in a hypotonic solution containing 1 × KHB and 0.4 mM EdCTP for 10 min. Then, the hypotonic solution was aspirated and the culture medium was added to the samples for 15 min. The procedure of cell extraction was adapted from Bennett *et al.* [29] and partly adjusted. EdC, EdU and their mono-, di- and triphosphates were analysed by the liquid chromatography system UltiMate 3000 (ThermoFisher Scientific) coupled with a Triple Quad 6500 mass spectrometer (Sciex). The chromatographic separations were performed at 35°C on a Luna NH2 (100 × 2.0 mm, 3 µm; Phenomenex) (for a detailed description, see the electronic supplementary material, S5).

### 2.11. Microscopy and data evaluation

The images were obtained by an Olympus IX81 microscope (objective: UPLFLN, 10× , NA 0.3) equipped with a Hamamatsu ORCA II camera with a resolution of 1344 × 1024 pixels using Cell^R acquisition software (Olympus) if not stated otherwise. For the acquisition of the high resolution images, a UPLANFLN, 40×, NA 1.3 objective was used. The data were analysed using CellProfiler image analysis software [30,31] and the final graphs were made in Microsoft Excel. For the evaluation of the MTT assay, Prism6 (GraphPad Software) was used.

All the measurements were performed for three independent experiments if not stated otherwise. For image cytometry, 10 000 cell nuclei were analysed per experiment. The data are presented as mean values ± s.e.m.

When the intensity signal of EdU and EdC was evaluated, we proceeded as follows if not stated otherwise.

At first, we determined from the histogram of average signal in cell nuclei the fraction of labelled cells (**F**, cells able to incorporate EdU or EdC) for the particular cell line and the time of incorporation. We analysed 10 000 cell nuclei in every sample. The cell nuclei were identified by DAPI staining. The analysis was performed using CellProfiler and Microsoft Excel software. For the evaluation, we used the average nuclear signal in the (**F**–0.1) 10 000 most-labelled nuclei (cells that contain the specific signal) and the signal in the (0.9–**F**) 10 000 least-labelled nuclei (cells without any specific signal). The average signal in the nuclei of the least-labelled cells was further subtracted from the average signal of the most-labelled cells. From the obtained value, we subtracted the signal of the cells incubated without the addition of EdU or EdC. This approach made it possible to minimize the impact of the variability of the background in the particular samples.

## 3. Results

### 3.1. EdC is converted to EdU in HeLa cells so effectively that it can be detected in nuclear DNA using an anti-bromodeoxyuridine antibody recognizing EdU

**Figure 1.**
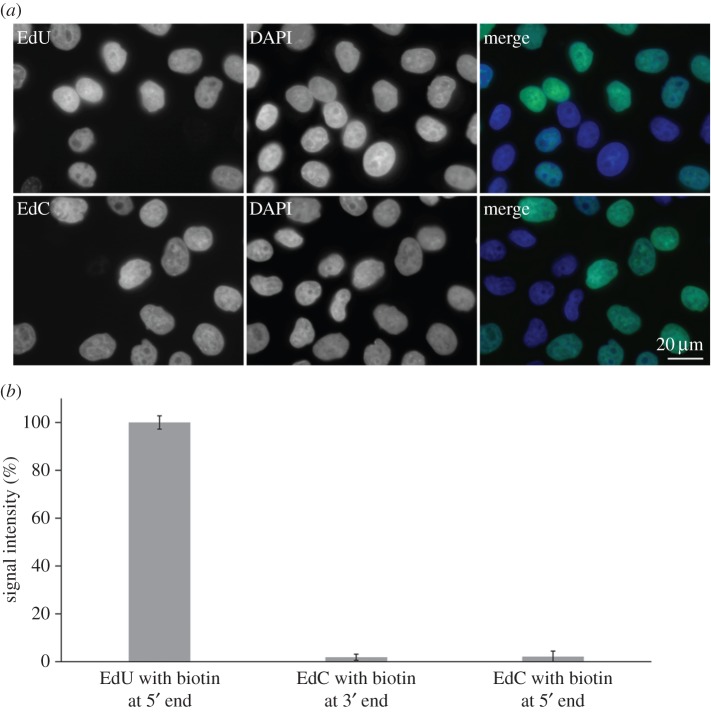
The microscopy analysis of EdC conversion to EdU using an antibody reaction. (*a*) Fluorescence detection of EdU by means of an anti-bromodeoxyuridine antibody (clone B44). HeLa cells were incubated with either 10 µM EdU or 10 µM EdC. Then, the detection of EdU (in green) and DNA using DAPI (in blue) was performed. (*b*) The analysis of the reactivity of anti-bromodeoxyuridine antibody (clone B44) using EdU with biotin at the 5′ end and EdC with biotin at the 3′ or 5′ end. The data were normalized to percentage of the signal provided by EdU (equal to 100%). The data are presented as mean ± s.e.m.

First, we tested whether EdC is converted to EdU using the detection of EdU in nuclear DNA. We supposed that if EdC is effectively converted to EdU, EdU will be further phosphorylated to EdUTP and subsequently incorporated into the DNA. We detected EdU by the anti-bromodeoxyuridine antibody (clone B44). This antibody has strong reactivity with EdU [26] but not with EdC (see below in this section). We incubated HeLa cells either with 10 µM EdU or EdC for 8 h and detected EdU in the nuclear DNA. In both cases, approximately 75% of the cells contained a signal corresponding to the localization of incorporated EdU (figure 1*a*). The ratio between the average signal in cells incubated with EdU and with EdC was 0.977 ± 0.091 (for details about the evaluation, see Material and methods, Microscopy and data evaluation section). The analysis of the signal was performed using image cytometry [12,19].

For the analysis of the reactivity of the anti-bromodeoxyuridine antibody clone B44 with EdU and EdC, we prepared biotinylated EdU and EdC and anchored them to the surface of the 96-well plates coated with streptavidin. Then, we incubated the well plates with primary anti-bromodeoxyuridine antibody and secondary antibody conjugated with Alexa Fluor 488 fluorochrome (see also [26]). As the affinity of anti-bromodeoxyuridine antibodies to BrdU depends on the position of the biotin coupling [12], we prepared EdC with the biotin both at 3′ and 5′ ends. In the case of EdU, the biotin was coupled to the 5′ end as this coupling results in a high signal [12]. The EdU-derived signal intensity after the subtraction of the negative control (the sample without EdU or EdC) was approximately 50 times higher than the signal produced by EdC and was nearly independent of the position of biotin (figure 1*b*).

Taken together, these results showed that HeLa cells converted EdC to EdUTP so effectively that the EdU produced can be detected in nuclear DNA by an antibody reaction.

### 3.2. EdC is not effectively incorporated into nuclear DNA

The tests performed with the antibody specifically reacting with EdU showed that EdC is converted into EdUTP that is then incorporated into DNA. However, this analysis did not reveal the extent of EdC incorporation. To address this issue, we incubated HeLa cells with 10 µM EdU or EdC for 8 h and subsequently we detected EdU and EdC in nuclear DNA using a click reaction. This system should produce a signal after the incorporation both of EdU and of EdC. If EdC is incorporated into the DNA, we should observe a decrease in the ratio between the signal in the nuclei of cells incubated with EdU and EdC after a click reaction as compared to the experiment in which we used an antibody specifically reacting with EdU. The average EdU- or EdC-derived signal in EdU- or EdC-positive nuclei was determined and then used for the determination of the ratio. For more details see Material and methods, Microscopy and data evaluation section. The ratio after the click reaction was 1.021 ± 0.127. As the ratio was almost the same as in the experiment with antibody detection (0.977 ± 0.091), EdU is probably a dominant nucleoside that is incorporated into the DNA of HeLa cells incubated with EdC.

**Figure 2.**
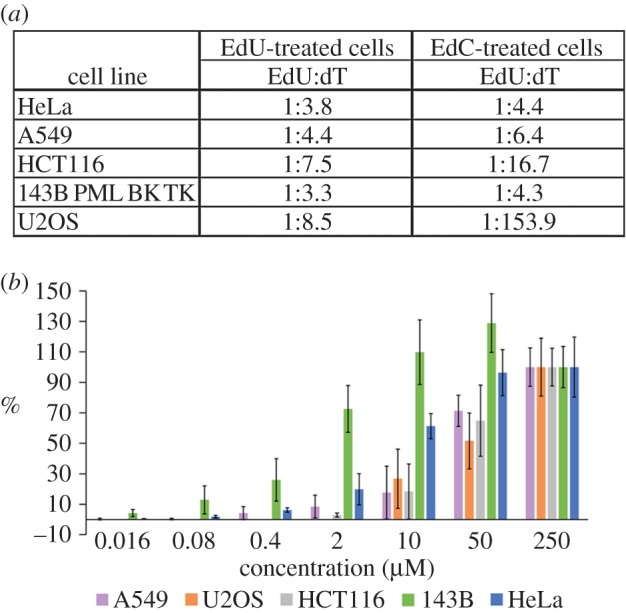
EdU- and dT-content ratios and the dependence of EdU incorporation on EdC concentrations. (*a*) The ratio between the content of EdU and dT in isolated DNA after a 24-h incubation with 10 µM EdU or EdC in five cell lines is shown. (*b*) The average nuclear signal in five cell lines incubated with 0.016–250 µM EdC for 4 h. The detection of the signal was performed using a click reaction. The data were normalized to the signal provided by 250 µM EdC (equal to 100%). The data are presented as mean ± s.e.m.

To exclude the possibility that the obtained data are a consequence of the approach used and/or dependent on the cell line used, we analysed the content of EdU, EdC and dT in five human cell lines incubated for 24 h with 10 µM EdU or EdC. After incubation with EdU or EdC or without any modified nucleosides (control cells), the DNA was isolated, cleaved by enzymes to produce mixtures of free nucleosides and the content of EdU, EdC and dT was analysed (figure 2*a*). In agreement with our finding in HeLa cells, all the tested cell lines contained EdU in their DNA. Surprisingly, not a single one of the DNA samples of the tested cell lines contained EdC. Simultaneously, we observed high differences in the amount of incorporated EdU among the particular cell lines.

To provide a more detailed description of the ability of the cell line to convert EdC to EdU and incorporate it into the DNA, we analysed the incorporation of EdU after a 4-h incubation of cells with various concentrations of EdC. We detected EdU using a click reaction (figure 2*b*). The highest ability to incorporate EdU was exhibited by the 143B PML BK TK (143B) cells, providing a signal in all the tested concentrations. HeLa cells provided a first weak signal with 0.08 µM EdC. The A549 cells had the first measurable signal in the case of 0.4 µM EdC, HCT116 at 2 µM EdC and U2OS cells at 10 µM EdC.

These results clearly showed that the incorporation of EdC is under the detection limit of the used methods. Simultaneously, it is obvious that the cell lines dramatically differ from one another in their ability to transform EdC to EdU and to incorporate it into the DNA. The highest ability to convert EdC to EdU and incorporate it into DNA was exhibited by 143B cells, the lowest ability was exhibited by U2OS cells.

### 3.3. The cytotoxicity of EdC is directly related to its conversion to EdU and its subsequent incorporation into DNA

The EdU toxicity is proportional to the level of EdU in DNA and it is supposed that it is related to the formation of interstrand cross-links [19]. EdUMP is also an inhibitor of thymidylate synthase [19,23]. This leads to an imbalance in the nucleoside and nucleotide pools, and due to the inhibition of dTMP synthesis also to the preferential incorporation of EdU into DNA [19].

In the case of EdC, its incorporation into DNA is extremely low, if there is any. With regard to our observation that EdC is converted to EdU which is subsequently incorporated into DNA, EdC toxicity might be largely mediated by EdU incorporation.

**Figure 3.**
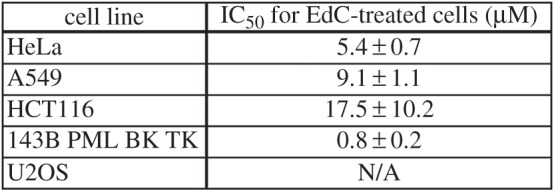
EdC toxicity in various cell lines. The measured IC_50_ values in cells influenced by EdC are shown. IC50 was determined from the results of MTT assay and calculated using the standard four parameter logistic nonlinear regression [19]. The data are presented as mean ± s.e.m.

We analysed EdC cytotoxicity using the MTT assay in five cell lines to clarify whether there is a relationship between the cytotoxic impact of EdC, its conversion to EdU and subsequent EdU incorporation into DNA. The sensitivity of particular cell lines to EdC significantly differed (figure 3). The most sensitive cells were 143B cells (half maximal inhibitory concentration, IC_50_ = 0.8 µM). The least sensitive were U2OS cells. In this case, we were not able to determine IC_50_ using nonlinear regression. However, because of the fact that U2OS surviving in the highest concentration of EdC was approximately 50%, we can suppose that the IC_50_ value is close to 250 µM.

It is apparent that the toxicity of EdC increases with the tendency to incorporate EdU into the DNA (cf. figure 2).

These results strongly indicated that the primary source of EdC cytotoxic effect is the conversion of EdC to EdU followed by EdU incorporation into the DNA.

### 3.4. Cytidine deaminase is a key deaminase in the pathway resulting in the production of EdUTP from EdC in HeLa cells

We tested the role of CDD and DCTD by means of their inhibition. While the specific siRNAs were used for inhibition of DCTD, the CDD activity was inhibited by specific siRNAs or by THU [24]. HeLa cells were used in these experiments as they can be effectively transfected with siRNAs, allow the reliable detection of cell nuclei using image cytometry and, concurrently, they have a relatively high ability to convert EdC to EdU and subsequently to incorporate it into the DNA.

**Figure 4.**
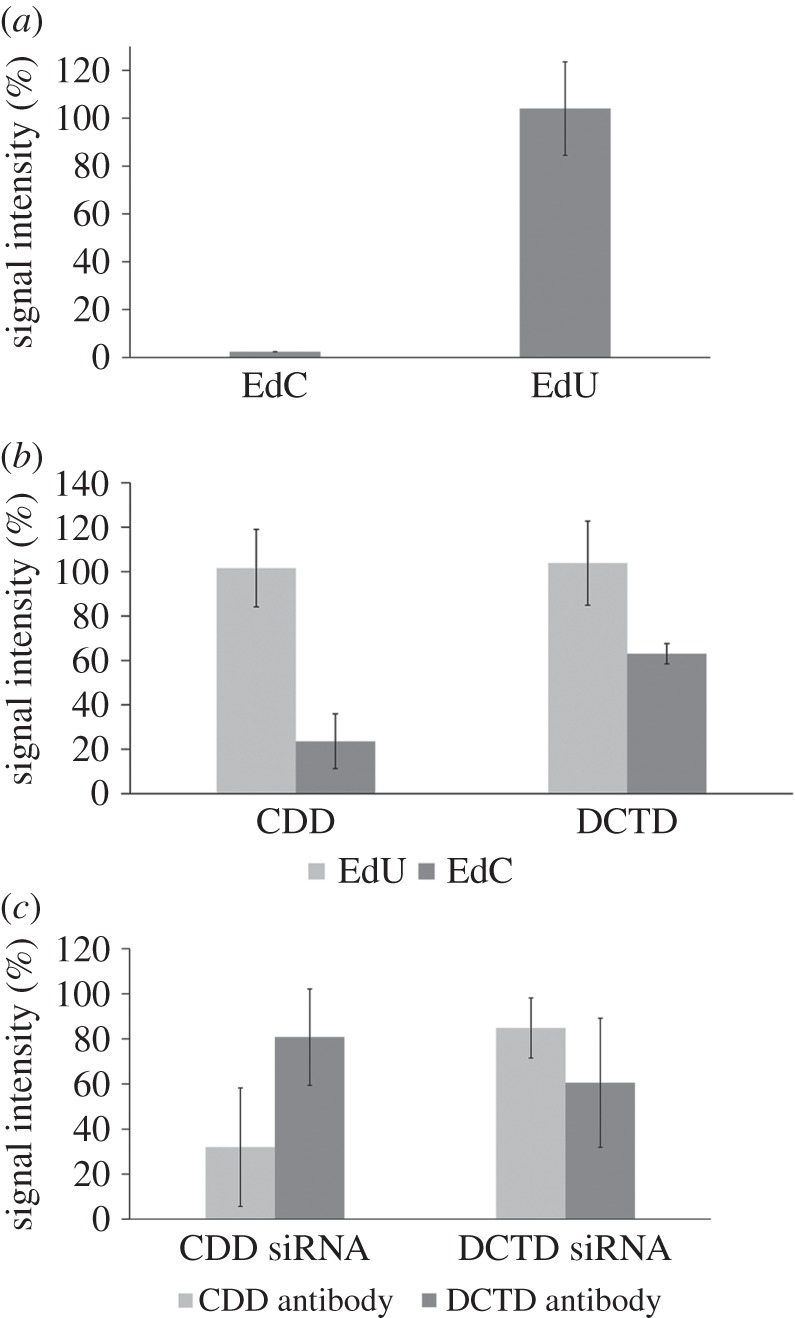
The impact of THU and specific siRNAs on the incorporation of EdU into DNA. (*a*) The EdU-derived signal intensity in HeLa cells incubated for 2 h with EdC or EdU in the presence of THU. For the detection of EdU, a click reaction was used. The data are normalized to percentage of the signal of control cells incubated with EdC or EdU without THU (equal to 100%, not shown). The data are presented as mean ± s.e.m. (*b*) The impact of siRNA against CDD and DCTD on the incorporation of EdU into the DNA in cells incubated for 2 h with EdU or EdC. For the detection of EdU, a click reaction was used. The data were normalized to percentage of the signal of cells incubated with control siRNA (equal to 100%, not shown). The data are presented as mean ± s.e.m. (*c*) The amount of CDD and DCTD measured by immunoblots in cells treated with siRNA against CDD and DCTD. The data were normalized to percentage of the signal of cells incubated with control siRNA (equal to 100%, not shown). The data are presented as mean ± s.e.m.

If THU was used, we first incubated cells for 30 min with 10 µM THU followed by a 2-h incubation in medium supplemented with 10 µM EdU or EdC and 10 µM THU. For the detection of EdU in DNA, we used a click reaction with azido fluorochrome. Approximately 45% of cells incubated with EdU exhibited a significant signal. We did not observe any impact of THU on the EdU signal (figure 4*a*). On the other hand, in the cells incubated with EdC, THU apparently almost completely stopped the deamination of EdC to EdU, as the incorporation of EdU into DNA was very low (figure 4*a*).

When we inhibited CDD activity by siRNA, we observed a decrease of the incorporation of EdU only in cells incubated for 2 h with EdC (figure 4*b*). The observed signal reached only approximately 24% of the signal in the control cells. The decrease of EdU incorporation was accompanied by a decrease of CDD expression (figure 4*c*). The expression of CDD was evaluated using immunoblots and corresponded to approximately 32% of CDD expression in control cells. It was in agreement with the data obtained from cells treated with THU. Accordingly, these results indicate that deamination mediated by CDD represents the major deamination route in the pathway from EdC to EdUTP.

In order to analyse the role of DCTD, we used siRNA against DCTD (figure 4*b*). In accordance with our expectation, it did not result in a decrease of EdU signal in the cells incubated with EdU. Surprisingly, in cells incubated with EdC, the signal decreased to approximately 63% of the signal in control cells. In addition, the expression of DCTD was decreased to approximately 61% of DCTD expression in control cells (figure 4*c*). It was a surprising observation with respect to the previous results and argues for an important role of DCTD at least under some circumstances.

Importantly, the siRNA used against CDD led also to a decrease of the expression of the DCTD and vice versa. While the siRNA primary against CDD decreased the expression of DCTD to approximately 81%, the siRNA against DCTD decreased the expression of CDD to approximately 60%. The performed analyses of nucleotide sequences of the used siRNAs and deaminases mRNA, however, did not show the possibility that it could be a consequence of sequence similarity.

Independently of the mechanism of this phenomenon, it is highly probable that the decrease of the signal of EdU caused by siRNA against DCTD can be attributed to the decrease of CDD and not to the decrease of DCTD. This is strongly supported by the nearly complete suppression of the signal with the CDD inhibitor THU in cells incubated with EdC, and relatively good correlation of the EdU signal and CDD content in cells incubated with EdC after the treatment with siRNA against CDD or DCTD.

### 3.5. Only a small fraction of EdC is converted into EdCTP when compared to the conversion of EdU to EdUTP

**Figure 5.**
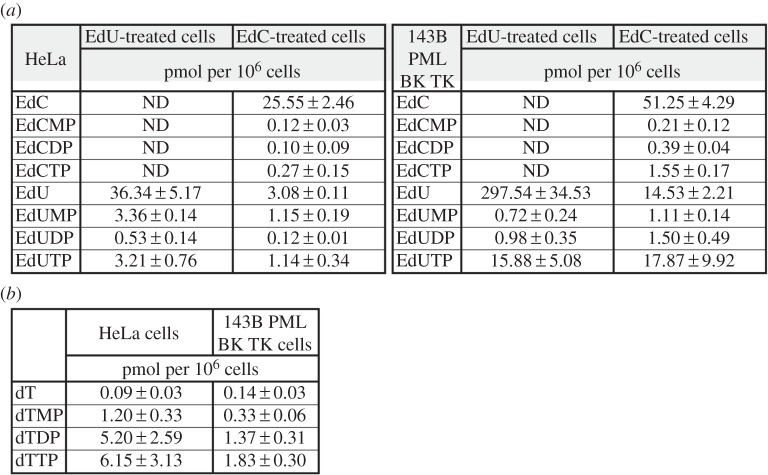
The content of EdU, EdC and their metabolites in HeLa cells incubated with EdU or EdC. (*a*) The amount of particular nucleosides and nucleotides in HeLa and 143B cells incubated with 10 µM EdU or EdC for 4 h. ND, non-detected. The data are presented as mean ± s.e.m. (*b*) The amount of thymidine and its nucleotides in HeLa and 143B cells. The data are presented as mean ± s.e.m.

Important data were provided by the analysis of the nucleoside and nucleotide pools. HeLa or 143B cells were incubated with 10 µM EdU or EdC followed by analysis of the concentration of nucleosides and nucleotides in the cells (figure 5*a*). A 4-h incubation was used. This time provided a bright EdU-derived signal and did not result in DNA damage preventing DNA replication. In addition, both EdC and EdU were present in the medium for the whole 4-h period as further prolongation of incubation time resulted in the increase of the fraction of labelled cells and also the signal intensity. In this respect, the time around 20 h resulted in the labelling of nearly all cells. It is in good agreement with the value of the doubling time of HeLa cells, which is around 18 h [19]. It strongly indicates that the incorporation can proceed for at least 8 h.

In the HeLa cells incubated with EdU, the highest portion was formed by EdU (approx. 84% of the sum of the intracellular concentrations of all the monitored nucleosides and nucleotides), EdUMP and EdUTP constituted around 7% and EdUDP around 1%. In the cells incubated with EdC, the percentage share of EdC was approximately 81% and EdU approximately 10%. Surprisingly, the percentage share of EdCTP was considerably lower than EdUTP. While the content of EdCTP was approximately 0.9%, the content of EdUTP was approximately 4%.

In the 143B cells incubated with EdU, the percentage share of EdU was around 94%, EdUMP constituted around 0.2%, EdUDP around 0.3% and EdUTP around 5%. In the cells incubated with EdC, the percentage share of EdC was approximately 58% and EdU approximately 16%. The percentage share of EdCTP was similar to that found in HeLa cells, considerably lower than EdUTP. While the content of EdCTP was approximately 1.8%, the content of EdUTP was approximately 20%.

Our analysis of thymidine and its monophosphate, diphosphate and triphosphate pools in non-treated HeLa and 143B cells (figure 5*b*) showed that the amount of EdUTP in EdU-treated cells was around 52% and 870%, respectively, of the measured dT concentration in the non-treated cells. The reasons for such large differences are not clear although the presence of viral thymidine kinase in 143B cells can be one of the factors playing an important role.

These data clearly showed that although EdC is converted to EdU, this conversion alone is not able to ‘protect’ the cell from the high levels of EdC and the subsequent incorporation of EdC into the DNA. Concurrently, they indicated that the conversion of EdC to EdCTP occurs much less effectively than the conversion of EdU to EdUTP. In this respect, we found in HeLa cells an approximately 12-fold (40-fold for 143B cells) lower level of EdCTP in the cells incubated with EdC compared with the amount of EdUTP in the cells incubated with EdU, although the EdC concentration was only 1.4 times (5.8-fold for 143B cells) lower than the concentration of EdU in the cells incubated with EdU. This finding points to the low effectivity of enzymes playing a role in the cascade leading to the formation of EdCTP and/or the high activity of enzymes mediating its degradation. In addition, the low level of EdCMP (around 0.4% or 0.2% in HeLa and 143B cells, respectively, of the sum of the intracellular concentrations of all the monitored nucleosides and nucleotides) in the cell incubated with EdC can substantiate the low role of DCTD in the pathway resulting in the conversion of EdC to EdUTP.

The analysis of EdUTP pools in 143B cells also indicated that the 10 µM EdC produced such an amount of EdU that resulted in the saturation level of EdUTP production. Moreover, our data indicate that the import of EdU can be much more efficient than the import of EdC in 143B cells (figure 5*a*). No such high difference was observed in the case of HeLa cells (figure 5*a*).

### 3.6. The replication complex serves as an important barrier of the incorporation of EdC into the DNA

To test the impact of the replication complex on the incorporation of EdC, we first used the run-on replication system. The system had been successfully used in the past for the detection of DNA replication by means of biotinylated dUTP (e.g. [32]).

**Figure 6.**
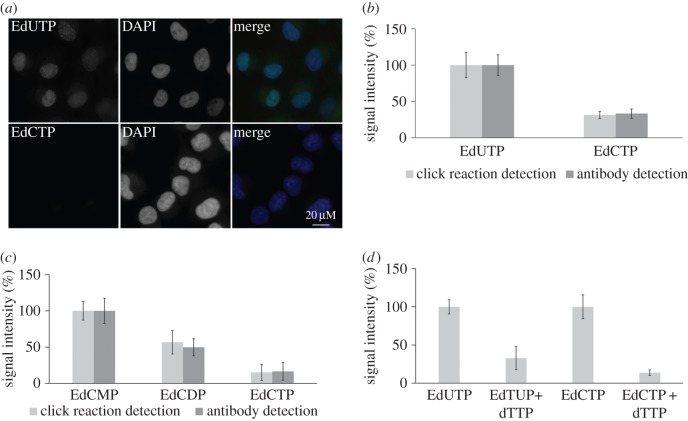
Run-on replication assay and hypotonic introduction of EdUTP and EdCTP, EdCDP and EdCMP. (*a*) The detection of EdU and EdC using Alexa Fluor 488 azide in permeabilized HeLa cells (in green). The nuclear DNA was stained by DAPI (in blue). (*b*) The average signal in cell nuclei after detection of EdU and EdC by Alexa Fluor 488 azide or EdU by the anti-bromodeoxyuridine antibody clone B44 in HeLa cells after the hypotonic introduction of EdUTP and EdCTP followed by a 30-min incubation in medium. The data are normalized to percentage of the signal of EdUTP-treated cells (equal to 100%). The data are presented as mean ± s.e.m. (*c*) The average signal in cell nuclei after detection of EdU and EdC by Alexa Fluor 488 azide or EdU by the anti-bromodeoxyuridine antibody clone B44 in HeLa cells after the hypotonic introduction of EdCMP, EdCDP and EdCTP followed by a 30-min incubation in medium. The data are normalized to percentage of the signal of EdCMP-treated cells (equal to 100%). The data are presented as mean ± s.e.m. (*d*) The average signal in cell nuclei after detection of EdU and EdC by Alexa Fluor 488 azide in HeLa cells after the hypotonic introduction of EdUTP or EdCTP with the concurrent introduction of dTTP. The data are normalized to percentage of the signal of EdUTP- or EdCTP-treated cells (equal to 100%). The data are presented as mean ± s.e.m.

The run-on assay was performed in the nucleotide mixture containing EdCTP or EdUTP. For the detection of these nucleotides we used a click reaction (figure 6*a*). While in the case of EdCTP we did not observe any significant signal, the application of EdUTP resulted in the labelling of around one-third of the cells. This result indicated that the replication complex and/or the repair mechanisms connected with replication are an effective barrier preventing the incorporation of EdC into DNA.

Alternatively, we used the HeLa cells with hypotonically introduced EdCTP or EdUTP. The hypotonic treatment has been successfully used in the past for the introduction of various low-molecular highly charged substances and in contrast with the permeabilized system does not lead to cell death [28,33]. The click reaction was used for the detection of EdU and EdC. The anti-bromodeoxyuridine antibody (clone B44) was used for the selective detection of EdU.

In all cases, we observed a clear nuclear signal (figure 6*b*). The observed signal was markedly stronger in cells incubated with EdUTP than with EdCTP. The signal in cells incubated with EdCTP reached approximately 30% of the signal measured in cells incubated with EdUTP for both detection systems. It indicated that the major portion of EdCTP was transformed to EdUTP, otherwise the signal strength provided by the click reaction (sum of the EdC- and EdU-derived signals) in cells incubated with EdC should be higher than the signal provided by an antibody (EdU-derived signal).

If EdCDP instead of EdCTP was introduced by hypotonic treatment, the signal increased (figure 6*b*). A further increase of the signal was observed when EdCMP was used (figure 6*c*). The gradual increase of the signal from EdCTP to EdCMP was the same in both detection systems (figure 6*c*). It was apparent that at least EdCTP and EdCDP were dephosphorylated and subsequently at the level of EdCMP or EdC deaminated and converted to EdU.

Next, we added also dTTP along with EdCTP or EdUTP in the hypotonic mixture (figure 6*d*). The detection of EdC and EdU was performed by a click reaction. In both cases, we observed a substantial decrease of the signal. If the cells were incubated with EdUTP and dTTP, the signal was decreased to approximately 32% of the signal from cells incubated with EdUTP alone. In the case of EdCTP and dTTP, the signal was decreased to 13% of the signal from cells incubated with EdCTP alone. This experiment further supported our previous results showing that EdC is not at all or only very unwillingly incorporated in nuclear DNA and that even EdCTP is gradually transformed into EdUTP.

**Figure 7.**
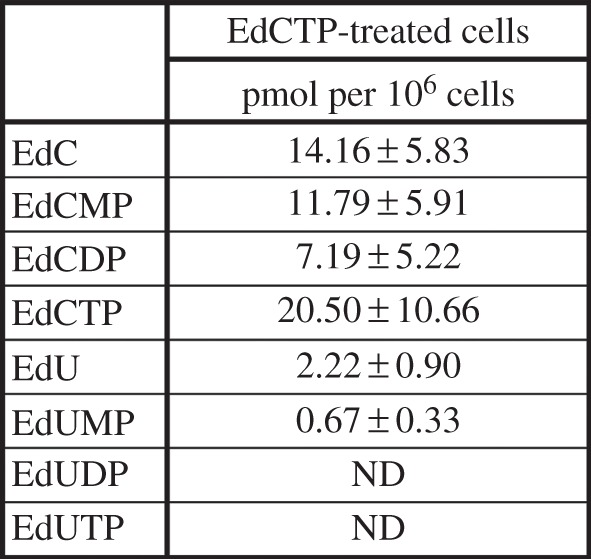
The content of EdCTP and its metabolites after introduction of EdCTP in HeLa cells. The amount of particular nucleosides and nucleotides in HeLa cells after the hypotonic introduction of 0.4 mM EdCTP followed by a 15-min incubation in medium. The concentration of EdUTP and EdUDP were under the detection limit of the method used. The data are presented as mean ± s.e.m.

The analysis of nucleotide and nucleoside pools in cells with hypotonically introduced EdCTP after a 15-min incubation in medium (figure 7) showed that they contained the highest concentration of EdCTP. The percentage share of the total content of metabolites of EdCTP were the following: EdCTP ∼ 30%, EdCDP ∼ 14%, EdCMP ∼ 23%, EdC ∼ 28%, EdU ∼ 4% and EdUMP ∼ 1%. Owing to the necessity to use high concentrations of EdCTP and the limited availability of this nucleotide, we were not able to process a sufficient number of cells for the determination of the concentration of EdUTP and EdUDP.

Hypotonic treatment led to approximately six times higher intracellular EdCTP concentration than was the concentration of EdUTP in cells incubated for 4 h with 10 µM EdU in HeLa cells. However, even such a high concentration was not sufficient for the considerable incorporation of EdC in the DNA. By contrast, the concentration of EdUTP, which was under the detection level in the hypotonically treated cells, allowed the effective detection of EdU in DNA.

The analysis of nucleoside and nucleotide pools also showed that enzymes with dephosphorylating activity play an important role in the EdU metabolism. In this respect, we observed its rapid dephosphorylation (the EdCTP to dephosphorylated products ratio was 3 : 7) already 15 min after the hypotonic introduction of EdCTP.

## 4. Discussion

In this study, we focused on the metabolism of two nucleosides commonly used as markers of cellular replicational activity—EdU and EdC. While in the case of EdC it was supposed to be incorporated into DNA instead of dC [5], EdU is incorporated into the DNA instead of dT [7].

Firstly, we addressed if EdC is effectively deaminated by cellular deaminases. The data from the experiments with a primary antibody reacting with EdU (clone B44) showed that EdC is effectively converted to EdU and this nucleoside analogue is incorporated into the DNA. Approximately 50 times higher ratio of the affinity of the used antibody to EdU than to EdC almost excludes the possibility that nearly the same signal in cells incubated with EdC and EdU is caused by exclusive EdC incorporation.

Much more surprising data were obtained by the comparison of the ratio between the nuclear signal in HeLa cells incubated either with EdU or EdC after detection with the clone B44 or by a click reaction and analysis of the content of EdU and EdC in the DNA of five cell lines incubated with EdC. The very similar ratios obtained suggested that there is no significant incorporation of EdC into the DNA, if any. The analysis of the EdC content in the DNA of the cells incubated with EdC confirmed this conclusion for the HeLa cells and showed that this is a more general phenomenon as none of the tested cell lines was able to incorporate EdC into their DNAs effectively. As far as the sensitivity of the HPLC method is concerned, it is evident that the possible EdC incorporation should be under the level corresponding approximately to one EdC to a thousand of dT.

This analysis also showed that all of the tested cell lines were able to deaminate EdC to EdU and incorporate this dT analogue into the DNA. The amount of incorporated EdU was cell line specific (figure 2). It indicates that the difference in the EdU incorporation in cells incubated with EdC is caused by the different deaminase activity resulting in the conversion of EdC to EdU. Apparently, the highest ability of EdC conversion and EdU incorporation was exhibited by 143B PML BK TK cells followed by HeLa cells, A549 cells, HCT116 cells and U2OS cells.

According to the published data, the EdC toxicity is lower than EdU toxicity [5]. While the EdU toxicity is supposed to be primarily caused by its incorporation into DNA and concurrently is amplified by inhibition of thymidylate synthase by EdUMP [19], the reasons of EdC toxicity were not well understood. As EdC is effectively converted to EdU which is subsequently incorporated into the DNA, a crucial part of the observed toxicity is in fact connected with the toxicity of the formed EdU. The results of the MTT assay showed that the toxicity mediated by this transformation is probably the most important contribution to EdC toxicity. The order of the cell lines with respect to their sensitivity to EdC (figure 3) was the same as their ability to convert EdC to EdU and subsequently to incorporate it into the DNA (figure 2).

According to our results, a fundamental role in EdC conversion in HeLa cells is played by CDD. The role of DCTD is probably only very marginal. This was strongly supported by the nearly complete suppression of the signal with the exclusive CDD inhibitor THU in cells incubated with EdC and a relatively good correlation of the intensity of EdU signal and the content of CDD in the cells incubated with EdC after treatment with siRNA against CDD or DCTD. Our non-published results with the inhibition of activity of CDD by THU showed that the CDD plays the most important role in the conversion of EdC also in HCT116 cells. Answering the question if this is a general phenomenon, however, will require additional experiments using a larger panel of various cell lines.

The activity of CDD is commonly considered to be one of the key factors that can influence the results of treatment when drugs based on the analogues of 2′-deoxycytidine (e.g. gemcitabine, cytarabine or decitabine) are used. These drugs are usually used for the treatment of adenocarcinomas, for the treatment of various solid tumours [34] or in the treatment of haematological malignancies [35]. It is known that gemcitabine is rapidly inactivated through deamination by CDD or in the monophosphate form by DCTD [36,37]. Notably, CDD has nearly half of the affinity for gemcitabine in comparison with 2′-deoxycytidine [38]. Cytarabine is a cytidine analogue; however, due to the ‘up’ orientation of the 2′-hydroxy group, it resembles the 2′-deoxycytidine structure [35]. Once incorporated into DNA, cytarabine results in the termination of the elongating, nascent DNA chain followed by cell death [39]. Cytarabine is, however, like gemcitabine, rapidly deaminated into 1-β-d-arabinofuranosyluracil by CDD with an initial plasma half-life of 7–20 min [40]. In this respect, Gandhi *et al.* [41] demonstrated the accumulation of ara-UTP in circulating blast cells of six patients with acute myeloid leukaemia (AML) treated with cytarabine. Another drug, decitabine, used for the treatment of myelodysplastic syndrome and AML, is also rapidly deaminated by CDD with a half-life of 15–25 min [42].

In contrast with the above-mentioned substances, the deamination of EdC leads to the production of the highly toxic product EdU. On the other hand, due to the possibility to follow EdU and EdC in DNA by means of click chemistry and EdU by antibodies, the EdC/EdU conversion is an interesting system for the analysis of its metabolism.

The measured EdUTP and EdCTP concentrations in cells incubated with EdC for 4 h indicated that the conversion of EdC to EdCTP is less effective in comparison to the conversion of EdU to EdUTP (figure 5*a*).

According to our results, the low production of EdCTP can be largely mediated by the dephosphorylation system of EdC phosphates. The experiments with the hypotonic introduction of EdCTP, EdCDP and EdCMP showed that the highest content of EdU in the DNA was exhibited by the cells incubated with EdCMP and the lowest content by cells incubated with EdCTP (figure 6*c*). Evidently, the progressive conversion of EdCTP to EdCDP, EdCMP and then either to EdC and subsequently to EdU by means of CDD or, directly, the conversion of EdCMP to EdUMP by DCTD occurred. The EdCTP degradation was surprisingly fast as the observed ratio of EdCTP to dephosphorylated products was 3 : 7 already 15 min after the hypotonic introduction of EdCTP.

In addition, the similar course of the dependence of the incorporation into DNA monitored by the EdU-specific antibody and click reaction after the hypotonic introduction of EdCTP indicated that not even an extremely high intracellular concentration of EdCTP leads to the effective incorporation of EdC into the DNA (figure 6*b*).

The fact that even a concentration of EdCTP substantially above the concentration of EdUTP in cells exhibiting a strong EdU signal did not result in DNA labelling by EdC indicated that the replication complex is unable to incorporate EdC into the DNA or EdC is quickly repaired. This conclusion is fully in agreement with the results of EdU and EdC incorporation in permeabilized cells. While in the case of the incubation of permeabilized cells with EdUTP we observed significant signal corresponding to the sites of EdU incorporation, the incubation with EdCTP did not provide any signal.

EdC is sometimes recommended instead of EdU for the labelling of nuclear DNA as its toxicity is lower than EdU. From our results it is evident that the use of EdC instead of EdU is quite controversial for four interconnected reasons:

(1) The obtained signal corresponds almost exclusively to the sites of EdU incorporation that were produced by the conversion of EdC.
(2) For the same level of the signal as EdU, a higher concentration of EdC has to be commonly used.
(3) As deamination of EdC may represent a limiting step of the EdC–EdUTP conversion, an extremely high concentration of EdC is sometimes necessary.
(4) The most important component of the EdC toxicity can be attributed to the incorporation of the produced EdU. Therefore, the signal increase is accompanied by an increase of its toxicity.

## Supplementary Material

Supplementary data
