# Supplementary material for: Dr Jekyll and Mr Hyde: a strange case of 5-ethynyl-2′-deoxyuridine and 5-ethynyl-2′-deoxycytidine
Source: Open Biol. 2016 Jan 6;6(1):150172. doi: 10.1098/rsob.150172 (PMC4736823; doi:10.1098/rsob.150172)
Supplement: Supplementary data [file rsob150172supp1.pdf]

SUPPLEMENTARY DATA

S1 Cell cultures

The HeLa cells were cultivated in Dulbecco's modified Eagle's medium (DMEM, Gibco) with 3.7 g/l of sodium bicarbonate, 143B PML BK TK cells (143B) in DMEM supplemented with 3.7 g/l of sodium bicarbonate and HAT (0.1 mM hypoxanthine, 400 nM aminopterin and 0.16 mM dT, Sigma Aldrich), A549 cells in F12K medium (Sigma Aldrich) with 1.5 g/l of sodium bicarbonate and HCT116 and U2OS cells in McCoy's medium (Sigma Aldrich) with 2.2 g/l of sodium bicarbonate if not stated otherwise. All the media were supplemented with 10% foetal bovine serum (Gibco), and 50 µg/ml of gentamicin. In the case of the 143B cells, we exchanged the culture medium for a HAT-free medium one week before the experiments. 143B cells containing viral TK were established by the transfection of 143B TK- cells with the vector containing pML-1 plasmid, sequence from the BK virus and hsv-1 TK gene. The transfected 143B PML BK TK cells stably express viral TK (Milanesi,G., et al. (1984) *Mol. Cell. Biol.* **4**, 1551-1560).

S2 Inhibition of CDD and DCTD activity

HeLa cells were grown in the 96-well plates in the culture medium without antibiotics for 24 hours, the cells were washed with the transfection medium (Santa Cruz Biotechnology) and 100 µl of transfection solution was added for 7 hours. The transfection solution consisted of 50 nM siRNA, transfection reagent and transfection medium (both, Santa Cruz Biotechnology). Then, 100 µl of the culture medium containing the double amount of foetal bovine serum and antibiotics was added. After 24 hours, the culture medium with the transfection solution was removed and the normal culture medium was added to the cells.

S3 DNA precipitation and denaturation

DNA was precipitated using 7.5 M ammonium acetate and ethanol (1 hour at -20°C). The samples were centrifuged (10,000 rpm, 10 minutes), DNA was two times washed with 75% ethanol, air-dried and dissolved in distilled water. The isolated and precipitated DNA was denaturated for 5 minutes at 100°C and cooled on ice for 15 minutes.

S4 Biotin-labelled EdC and EdU preparation

Briefly, the 2′-deoxy-5′-*O*-dimethoxytrityl-5-ethynyl-3′-*O*-uridinyl hemisuccinate, 2′-deoxy-3′-*O*-dimethoxytrityl-5-ethynyl-5′-*O*-uridinyl hemisuccinate, 2′-deoxy-4-*N*-dibutylaminomethylene-5′-*O*-dimethoxytrityl-5-ethynyl-3′-*O*-cytidinyl hemisuccinate, and 2′-deoxy-4-*N*-dibutylaminomethylene-3′-*O*-dimethoxytrityl-5-ethynyl-5′-*O*-cytidinyl hemisuccinate were attached to the solid support (Amino-SynBase™ CPG 500/110) and, after detritylation, coupled with 1-dimethoxytrityloxy-3-*O*-(*N*-biotinyl-3-aminopropyl)-triethyleneglycolyl-glyceryl-2-*O*-(2-cyanoethyl)-(*N*,*N*-diisopropyl)-phosphoramidite (Glen Research) using the standard phosphoramidite trityl-on protocol of the oligonucleotide synthesis. The desired protected products were released from the solid support by treatment with pressured gaseous ammonia (100 psi, 2 h, r.t.), the solid support was washed with 75% aq. acetic acid (1.5 ml); the effluent was set aside at r.t. for 30 min to remove the dimethoxytrityl group and then lyophylised. The crude biotinylated EdC and EdU derivatives were purified on the reversed phase column (Luna C18, 5 µm, 10×250 mm, 3 ml/min, gradient 0→50% acetonitrile in 0.05 M-triethylammonium hydrogen carbonate) using an LCMS device (Autopurification System, Waters). Purified compounds were lyophylised.

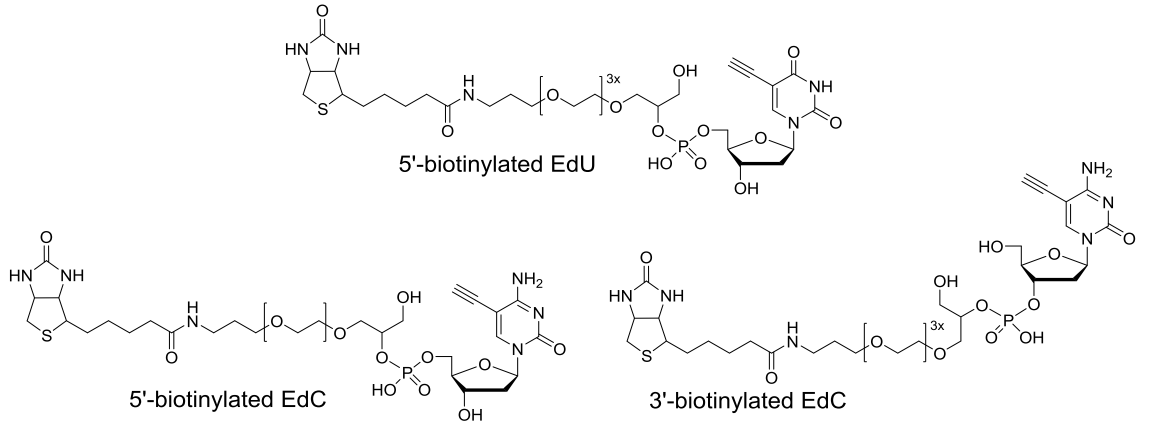

S5 Nucleotide pools analysis

Cells treated with EdU or EdC or without any treatment in medium were immediately and quickly vacuum filtered. Membrane filter was transferred to pre-cooled Petri dish and 2.5 ml of cold extraction solution (AcCN : MetOH : 0.5M FA/ 30:10:10) was added. Dishes were returned to the -20°C freezer for 30 minutes. The filter was rinsed by extraction solution in the dish and then the solution was transferred into two centrifuge microtubes. 1 ml of fresh cold extraction solution was used for re-rinsing the filter, resulting solution was added to the initial cell extract in the microtubes. Extraction solution was centrifuged (16,000 x g, 4°C, 5 minutes), supernatant was transferred into clean conical tube and put back into the freezer. Pellet was resuspended in 100 µl of extraction solution, remained for 15 minutes at -20°C, centrifuged and supernatant was mixed with the original supernatant from the previous step. The extract was neutralized with 300 µl of 15% NH_4HCO_3 and centrifuged. The supernatant was lyophilized and resuspended in 100 µl of mobile phase before the mass spectrometry analysis. The mobile phase consisted of 20 mM ammonium acetate, pH = 9.75 (A) and acetonitrile (B). During first seven minutes, the linear gradient from 95 to 10 % of B was applied, in the next seven minutes the mobile phase consisted of 10 % of B. Then the system was set to initial conditions (95 % B) in one minute and held for the next three minutes. The flow rate was set at 0.3 mL/min and the total analysis time was 18 min. All the experiments were performed on a triple quadrupole mass spectrometer with electrospray ionization in both positive and negative mode. The mass spectrometer was operated in multiple reaction monitoring (MRM) mode with a dwell time of 10 ms for EdC and EdU and to 50 msec for all nucleotide phosphates. The parameters of the ion source were optimized to the following settings: an ionization spray voltage of 4500 V and -4500 for positive and negative mode, respectively, a curtain gas of 30 psi, a collision gas of 8 psi, a heater gas and turbo ion spray gas of 40 psi, a source temperature of 400 °C, and an entrance potential of (-) 10 V. High-purity nitrogen was used as the collision gas. The declustering potential, collision energy, and collision cell exit potential were optimized on standards of the analytes being studied in a solution of mobile phase A:B = 50:50 (v/v). All the parameters are detailed in Table 1. Both quadrupoles (Q1 and Q3) were set to “unit” mass resolution. The Analyst 1.6.2 and MultiQuant 3.0 software (Sciex, USA) were used for data acquisition and evaluation. The EdUMP and EdUDP standards were not available and mass spectrometry conditions were adopted from optimisation experiments of the EdCMP/EdCDP/EdCTP standards. In order to quantify the peak areas of EdCMP and EdCDP related to EdCTP/EdUTP, ratio were used.

Table 1 Optimized mass spectrometry parameters for the analysed compounds.

Mode	Q1	Q3	Analyte	DP (V)	CE (V)
positive	252.0	136.1	EdC	46	15
	332.0	136.1	EdCMP	36	17
	**412.0**	**216.1**	**EdCDP**	**51**	**13**
	**491.9**	**136.1**	**EdCTP**	**71**	**19**
	253.0	137.1	EdU	116	17
	333.0	81.1	EdUMP	36	53
	492.9	81.0	EdUTP	111	55
negative	**249.9**	**136.0**	**EdC**	**-55**	**-12**
	**329.9**	**194.8**	**EdCMP**	**-55**	**-22**
	409.9	274.7	EdCDP	-45	-26
	489.8	158.8	EdCTP	-50	-34
	**250.9**	**134.8**	**EdU**	**-50**	**-16**
	**331.0**	**135.0**	**EdUMP**	**-55**	**-26**
	**411.0**	**78.9**	**EdUDP**	**-70**	**-102**
	**490.9**	**158.8**	**EdUTP**	**-70**	**-34**

DP – declustering potential
EP – entrance potential
CE – collision energy
CXP – collision cell exit potential
*bold *m/z* transitions were used for quantitation
